# Supplementary material for: Comparison of Outcomes after Arthroscopic Rotator Cuff Repair between Elderly and Younger Patient Groups: A Systematic Review and Meta-Analysis of Comparative Studies
Source: Diagnostics (Basel). 2023 May 17;13(10):1770. doi: 10.3390/diagnostics13101770 (PMC10217625; doi:10.3390/diagnostics13101770)
Supplement: Supplementary file 1 [file diagnostics-13-01770-s001.zip › diagnostics-2350733-supplementary/File S1_search strategy.pdf]

Appendix 1. MEDLINE (Ovid) search strategy (13th Sep 2022)

1 shoulder/
2 rotator cuff/
3 1 or 2
4 calcium/
5 exp bursitis/
6 4 or 5
7 3 and 6
8 shoulder pain/
9 shoulder impingement syndrome/
10 rotator cuff injuries/
11 (rotator cuff or supraspinatus or infraspinatus or subscapular$ or teres).tw.
12 ((shoulder$ or subacromial or rotator cuff) adj5 (tendon$ or tendin$ or bursitis or calcium or calcif$ or impinge$ or tear$ or pain)).tw.
13 or/7-12
14 exp Surgical Procedures, Operative/
15 su.fs.
16 (surger$ or surgical$ or operat$).tw.
17 decompress$.tw.
18 bursectom$.tw.
19 acromioplast$.tw.
20 (calcium adj remov$).tw.
21 debrid$.tw.
22 ARTHROSCOPY/
23 arthroscop$.tw.
24 or/14-23
25 13 and 24
26 age. mp.
27 (seventy or “70”).tw.
28 and/25-27 (818)

Appendix 2 Embase (Elsevier) search strategy (13th Sep 2022)

#1 'shoulder'/exp
#2 'rotator cuff'/exp
#3 #1 or #2
#4 'calcium'/exp
#5 'bursitis'/exp
#6 #4 or #5
#7 #3 and #6
#8 'shoulder pain'
#9 'shoulder impingement syndrome'/exp
#10 'rotator cuff injury'/exp
#11 (rotator cuff or supraspinatus or infraspinatus or subscapular$ or teres):ab,ti
#12 (shoulder$ or subacromial or 'rotator cuff') NEAR/5 (tendon$ or tendin$ or bursitis or calcium or calcif$ or impinge$ or tear$ or pain)
#13 #7 OR #8 OR #9 OR #10 OR #11 OR #12
#14 'surgery'/exp
#15 (surger$ or surgical$ or operat$):ab,ti
#16 decompress$:ab,ti
#17 bursectom$:ab,ti
#18 acromioplast$:ab,ti
#19 (calcium NEXT/10 remov$):ab,ti
#20 'debridement'
#21 debrid$:ab,ti
#22 'shoulder arthroscopy'/
#23 arthroscop$:ab,ti
#24 #14 OR #15 OR #16 OR #17 OR #18 OR #19 OR #20 OR #21 OR #22 OR #23
#25 #13 and #24
#26 'age'
#27 seventy or 70
#28 #25 and #26 and #27
#29 #28 AND [embase]/lim NOT ([embase]/lim AND [medline]/lim) (301)

Appendix 3. CENTRAL (Cochrane Library) search strategy (13th Sep 2022)

#1 MeSH descriptor: [Shoulder] this term only
#2 MeSH descriptor: [Rotator Cuff] this term only
#3 #1 or #2
#4 MeSH descriptor: [Calcium] this term only
#5 MeSH descriptor: [Bursitis] 1 tree(s) exploded
#6 #4 or #5
#7 #3 and #6
#8 MeSH descriptor: [Shoulder Pain] this term only
#9 MeSH descriptor: [Shoulder Impingement Syndrome] this term only
#10 MeSH descriptor: [Rotator Cuff Injuries] this term only
#11 rotator cuff:ti,ab or supraspinatus:ti,ab or infraspinatus:ti,ab or subscapular*:ti,ab or teres:ti,ab
#12 ((shoulder*:ti,ab or subacromial:ti,ab or rotator cuff:ti,ab) near/5 (tendon*:ti,ab or tendin*:ti,ab or bursitis:ti,ab or calcium:ti,ab or calcif*:ti,ab or impinge*:ti,ab or tear*:ti,ab or pain:ti,ab))
#13 #7 or #8 or #9 or #10 or #11 or #12
#14 MeSH descriptor: [Surgical Procedures, Operative] explode all trees
#15 (surger*:ti,ab or surgical*:ti,ab or operat*:ti,ab)
#16 decompress*:ti,ab
#17 bursectom*:ti,ab
#18 acromioplast*:ti,ab
#19 (calcium:ti,ab next remov*:ti,ab)
#20 debrid*:ti,ab
#21 MeSH descriptor: [Arthroscopy] this term only
#22 arthroscop*:ti,ab
#23 #14 or #15 or #16 or #17 or #18 or #19 or #20 or #21 or #22
#24 #13 and #23
#25 age*:ti,ab
#26 (seventy or “70”):ab,ti
#27 #24 and #25 and #26 (127)
#28 #27 (Trials) 124

818+301+124→ 1185 (58 duplicate)
