# Supplementary material for: Comparison of Outcomes after Arthroscopic Rotator Cuff Repair between Elderly and Younger Patient Groups: A Systematic Review and Meta-Analysis of Comparative Studies
Source: Diagnostics (Basel). 2023 May 17;13(10):1770. doi: 10.3390/diagnostics13101770 (PMC10217625; doi:10.3390/diagnostics13101770)
Supplement: Supplementary file 1 [file diagnostics-13-01770-s001.zip › diagnostics-2350733-supplementary/File S2 excluded studies.pdf]

File S2. Excluded studies with reasons

Study	Reference	Reasons for exclusion
Worland 1999	Repair of massive rotator cuff tears in patients older than 70 years https://pubmed.ncbi.nlm.nih.gov/10077792/	Case series
Charousset 2010	Arthroscopic repair of full-thickness rotator cuff tears: is there tendon healing in patients aged 65 years or older? https://pubmed.ncbi.nlm.nih.gov/20206038/	Case series
Verma 2010	Outcomes of arthroscopic rotator cuff repair in patients aged 70 years or older https://pubmed.ncbi.nlm.nih.gov/20729024/	Case series
Cozzolino 2012	Outcome of single tendon rotator cuff repair in patients older than 65 years https://pubmed.ncbi.nlm.nih.gov/22743069/	Conference abstract, case series
De Carvalho 2012	Open rotator cuff repairs in patients 70 years and older https://pubmed.ncbi.nlm.nih.gov/22519638/	case series
Djahangirl 2013	Outcome of single-tendon rotator cuff repair in patients aged older than 65 years https://pubmed.ncbi.nlm.nih.gov/22743069/	case series
Flurin 2013a	Rotator cuff tears after 70 years of age: a prospective, randomized, comparative study between decompression and arthroscopic repair in 154 patients https://pubmed.ncbi.nlm.nih.gov/24211128/	Not planned comparison
Flurin 2013b	Arthroscopic repair of the rotator cuff: prospective study of tendon healing after 70 years of age in 145 patients https://pubmed.ncbi.nlm.nih.gov/24200997/	Not planned comparison
Robinson 2013	Rotator cuff repair in patients over 70 years of age: early outcomes and risk factors associated with re-tear https://pubmed.ncbi.nlm.nih.gov/23365029/	Case series
Bhatia 2015a	Two-year outcomes following arthroscopic rotator cuff repair in recreational athletes over 70 years of age	Conference abstract, case series
Bhatia 2015b	Two-Year Outcomes After Arthroscopic Rotator	Conference abstract, case

	Cuff Repair in Recreational Athletes Older Than 70 Years https://pubmed.ncbi.nlm.nih.gov/25834140/	series
Bhatia 2015c	Two-year outcomes following arthroscopic rotator cuff repair in recreational athletes over 70 years of age https://pubmed.ncbi.nlm.nih.gov/25834140/	Case series
De Castro 2015	Rotator cuff injury in patients over the age of 65 years: evaluation of function, integrity and strength https://pubmed.ncbi.nlm.nih.gov/26229937/	Case series
Miyazaki 2015	Evaluation of the results from arthroscopic surgical treatment of rotator cuff injuries in patients aged 65 years and over https://pubmed.ncbi.nlm.nih.gov/26229935/	Case series
Silva 2017	Surgical Treatment of Rotator Cuff Tears After 65 Years of Age: A Systematic Review https://pubmed.ncbi.nlm.nih.gov/28555558/	Review
Kwon 2019	The Rotator Cuff Healing Index: A New Scoring System to Predict Rotator Cuff Healing After Surgical Repair https://pubmed.ncbi.nlm.nih.gov/30485753/	irrelevant
Witney-Lagen 2019	Do elderly patients gain as much benefit from arthroscopic rotator cuff repair as their younger peers? https://pubmed.ncbi.nlm.nih.gov/30704915/	Not planned comparison
Altintas 2020	Repair of Rotator Cuff Tears in the Elderly: Does It Make Sense? A Systematic Review https://pubmed.ncbi.nlm.nih.gov/31038992/	Review
Stone 2020	Midterm outcomes of arthroscopic rotator cuff repair in patients aged 75 years and older https://pubmed.ncbi.nlm.nih.gov/32088076/	Case series
