# Supplementary material for: Comparison of Outcomes after Arthroscopic Rotator Cuff Repair between Elderly and Younger Patient Groups: A Systematic Review and Meta-Analysis of Comparative Studies
Source: Diagnostics (Basel). 2023 May 17;13(10):1770. doi: 10.3390/diagnostics13101770 (PMC10217625; doi:10.3390/diagnostics13101770)
Supplement: Supplementary file 1 [file diagnostics-13-01770-s001.zip › diagnostics-2350733-supplementary/File S3 Quality of included studies.pdf]

File S3 Quality of included studies

	Selection				Comparability	Outcome			Total
Cohort studies									
Study (author, year)	Representativeness of the exposed cohort	Selection of the non-exposed cohort	Ascertainment of exposure	Demonstration that outcome of interest was not present at start of study	Comparability of cases and controls on the basis of the design or analysis	Assessment of outcome	Was follow-up long enough for outcomes to occur	Adequacy of follow up of cohorts	
Osti, 2010*	✵	✵	✵	✵	✵	--	✵	✵	7
Rhee, 2014*	✵	✵	✵	✵	✵	--	✵	✵	7
Moraiti, 2015*	✵	✵	✵	✵	✵	--	✵	✵	7
Nicloson, 2019*	✵	✵	✵	✵	✵	--	✵	✵	7

Case-control study

	Selection				Comparability	Exposure			Total
	Is the case definition adequate?	Representative-ness of the cases	Selection of Controls	Definition of Controls	Comparability of cases and controls on the basis of the design or analysis	Ascertain-ment of exposure	Same method of ascertainment for cases and controls	Non-Response rate	
Gwark, 2018**	✵	✵	--	✵	✵✵	✵	✵	--	7

*Quality assessed by Newcastle-Ottawa quality assessment scale for cohort study

** Quality assessed by Newcastle-Ottawa quality assessment scale for case-control study
